# Book Review: Difficult Subjects: Insights and Strategies for Teaching About Race, Sexuality, and Gender

**DOI:** 10.3389/fsoc.2020.00025

**Published:** 2020-04-15

**Authors:** Hannah Terese Whitley

**Affiliations:** Department of Agricultural Economics, Sociology, and Education, Pennsylvania State University, University Park, PA, United States

**Keywords:** difficult subjects, gender, race, sexuality, teaching strategies

Badia Ahad-Legardy and OiYan A. Poon (Sterling, VA: Stylus Publishing, LLC), 2018, 304 pages, ISBN:978-1-62036-792-6

“Teaching is hard,” writes Lori Patton Davis. “The act of teaching subjects such as race, gender, and sexuality, and doing so with a level of critical consciousness, thoughtfulness, and care presents even greater difficulty” [(Ahad-Legardy and Poon, 2018), p. ix]. High-quality teaching and learning in the twenty-first century require resistance to dehumanization, standardization, and corporatization (hooks, 2003; Berg and Seeber, 2016). At the same time, however, confronting racism, sexism, classism, homophobia, transphobia, and similar discriminations within the classroom requires an uncomfortable recognition of unequal privileges that counter national narratives of life, liberty, happiness, and equal opportunity for achieving the “American dream.” Teaching in ways that confront difficult subjects presents even more challenges for college educators, especially those with marginalized identities or professionally vulnerable positions such as contingent or pre-tenure faculty. Notably, women and faculty of color are disproportionally represented among contingent faculty (Finley, 2009). In their timely and overdue guide for educators, co-editors Badia Ahad-Legardy and OiYan A. Poon provide a roadmap for assisting post-secondary teachers in navigating the tumultuous terrain of difficult classroom dialogues in the United States.

As Ahad-Legardy and Poon write in their closing remarks, this book, which “was in progress well-before the election of Donald Trump to the U.S. presidency” (p. 267), was produced in part as a response to the cultural moments of terrorist attacks, police shootings, #BlackLivesMatter, the Syrian civil war, and college protests that have shaped our world over the past 10 years. Written by contributors who teach in post-secondary classrooms in the United States, *Difficult Subjects* is a collection of essays from scholars across disciplines, institutions, and ranks. Most authors come to their topic from a clearly articulated left-leaning perspective. Frequent references are made to the “neoliberal university” and its challenges for educators dedicated to helping students think critically about race, gender, and sexuality across disciplines. Throughout the volume, contributors share diverse and multifaceted approaches to teaching about subjects that prove challenging and often uncomfortable for the professor and student alike. Authors expand on these challenges through thoughtful narratives that highlight questions regarding positionality, the need for educators to disrupt the status quo in the curriculum, how different identities and bodies are read in the classroom, and relevant strategies for dealing with challenges as they emerge. Ahad-Legardy and Poon have organized *Difficult Subjects*' 16 chapters into three sections arranged by theme. Each chapter explores the realities associated with difficult dialogues and offers an avenue through which readers across a range of disciplines and institution types gain a more comprehensive understanding of thoughtful and equitable teaching processes.

Contributors in Part One, “(Dis)comfort, Fragility, and the Intersections of Identity,” explore notions and challenges of comfort and discomfort in the classroom. Essays in this section address the struggles (and successes) of encouraging discomfort as a necessary aspect of the learning process and explore how the notion of comfort has been deployed to avoid or deflect from engaging anti-racist pedagogies. As a white woman sociologist looking for ways to utilize these frameworks within my own classes, the chapter that most engaged me in Part One was Ambikar, Guentchev, and Lunt's “A Conversation on Challenging and Using Comfort-Zone Racism in the Classroom” (p. 19–34). In this chapter, the authors—Ambikar, an Indian woman, Guentchev, a white man from Bulgaria, and Lunt, a white man from the United States—present self-reflective analyses of how their personal identities interact with student perceptions and how keeping those interaction effects in mind have lead to specific teaching strategies. The chapter argues for co-teaching as an effective strategy for addressing comfort-zone racism and considers how the racialized and ethnic identities of professors can be used, rather than ignored or rendered neutral, to facilitate anti-racist pedagogies. Ambikar, for example, describes one assignment she assigns in an introductory class about globalization. During one class meeting, she describes the global competition that students will face in their own job searches and asks them if she, an immigrant woman, should be at a U.S. university teaching them. She writes, “After some humor and cajoling, one or two students will usually say ‘no’ and admit they feel an American, which is often popularly understood as a White American… should hold [her] job. This answer helps the students identify the anti-immigrant or unconscious bias that may be prevalent in class” (p. 25), and it is at this point that she asks students to produce self-reflective journal entries which helps them explicitly identify their own unconscious biases—about race, immigration, and gender.

Part Two, “Embracing Embodiment and Emotion as Pedagogical Praxis,” features essays that center human emotion, experience, and affect as crucial components to powerful pedagogies. The chapters in this part challenge conventional pedagogical logic by placing the queer, raced, gendered body in the center of classroom conversations, and engaging emotion and/or affect as a form of intellectual inquiry. Adriana Estill's chapter, “Feeling Our Way to Knowing: Decolonizing the American Studies Classroom” (p. 113–128), for example, raises important questions of how white students, in particular, confront and engage with contemporary realities of racism and other forms of oppression and violence faced by non-white people. Estill offers several concrete pedagogical tools, such as the creation of group-developed contracts, participation in the fishbowl discussion circle, and acknowledging, not ignoring, the emotions of raced/queer/marginalized students. “In order for [students'] emotion to be understood as productive knowledge-making,” Estill writes, “the presumed neutral knowing of the White students must be unbalanced and questioned” (p. 125). Estill explains that not looking away from marginalized students' anger, frustration, and emotion in the classroom “means recognizing White ignorance and making a commitment to remembering” (p. 125), thus demonstrating how embracing emotionality for knowledge can disrupt hegemonic whiteness in post-secondary classrooms.

Lastly, the chapters in Part Three, “Radical Pedagogy in ‘Neutral’ Places,” bring attention to teaching difficult subjects in unexpected fields like STEM disciplines, which are often presumed objective or politically neutral. While the majority of chapters in this volume highlight ways that faculty can make the classroom and campus environment amenable to difficult conversations, Jasmine L. Harris argues in “Uncomfortable Learning: Teaching Race Through Discomfort in Higher Education” (p. 248–265) that pedagogies focused on safe and brave spaces do not adequately address how faculty can productively make use of discomfort. Harris defines “safe spaces” and “brave spaces” but goes on to challenge them as “raced and gendered” (p. 252), offering an alternative in the practice and cultivation of an Uncomfortable Learning Approach, which loosely follows Bloom's taxonomy using the tenets of remembering; understanding; applying, analyzing, and evaluating; and synthesizing as progressively challenging modes of learning. Through the Uncomfortable Learning Approach, students dissect the visceral experience of discomfort by engaging in critical classroom discussions on issues of race and racism from a diverse set of viewpoints. As a pedagogical tool, this approach acts as a “kind of educational therapy, using intense emotional responses to facilitate learning” (p. 259). Referencing a case study conducted at a predominantly white U.S. university, Harris describes how student emotional responses led to deep learning and emotional breakthroughs from individuals who were used to ignoring the experience of emotions in the classroom (p. 260). As a result, Harris suggests that the Uncomfortable Learning Approach be adapted into classrooms engaging with difficult subjects, as negative emotions can be powerful tools in advancing a socially just and equitable pedagogy.

*Difficult Subjects* is an accessible guide for educators dedicated to teaching and learning within the framework of social justice and interdisciplinarity, and with an eye to the current political and cultural climate in the United States. Though written with a post-secondary audience in mind, the volume is well-situated to offer practical pedagogical tips for high school faculty who are committed to tackling difficult conversations in the classroom and digging deeper into issues surrounding race, gender, and sexuality. Providing practical advice and best practices that address the needs of all instructors regardless of experience, *Difficult Subjects* is an essential read for graduate students and recent PhDs. The volume may also be an inspiration for instructors who believe their area of study does not allow for such pedagogical inquiries to teach in ways that address difficult subjects. Importantly, *Difficult Subjects* fills a significant gap in the literature on post-secondary education by encouraging college educators to engage in pedagogy that does not pretend teachers and students are unaffected by world events and incidents that highlight social inequities.

## Author Contributions

HW conceptualized and designed the book review, wrote all sections of the manuscript, read, and approved the submitted version.

### Conflict of Interest

The author declares that the research was conducted in the absence of any commercial or financial relationships that could be construed as a potential conflict of interest.
